# Vitamin D status at birth among pregnant women and their newborns in Jordan: a cross-sectional study

**DOI:** 10.3389/fmed.2026.1864210

**Published:** 2026-06-09

**Authors:** Rawan A. Obeidat, Baraa Sakee, Shahd Alguzo, Sakhr Alshwayyat, Omar F. Altal, Hasan Rawashdeh

**Affiliations:** 1 Department of Obstetrics and Gynecology, Faculty of Medicine, Jordan University of Science and Technology, Irbid, Jordan; 2 Faculty of Medicine, Jordan University of Science and Technology, Irbid, Jordan

**Keywords:** birth weight, cord blood, newborn, pregnancy, supplementation, vitamin D

## Abstract

**Background:**

Growing evidence suggests a link between vitamin D deficiency during pregnancy and an increased risk of adverse pregnancy and birth outcomes.

**Objective:**

This study aimed to measure serum 25-hydroxyvitamin D (25[OH]D) concentrations in pregnant women and their newborns and to explore potential associations between vitamin D levels and maternal and neonatal factors.

**Methods:**

This cross-sectional study was conducted at King Abdullah University Hospital (KAUH) in Jordan. A total of 396 pregnant women who gave birth between November 2021 and July 2022 were recruited. Maternal and cord blood samples were collected at the time of delivery, and serum vitamin D concentrations were measured using an enzyme-linked immunosorbent assay (ELISA). The Mann–Whitney U test and Spearman’s correlation test were used for statistical analysis. A multivariable linear regression analysis was performed to identify independent predictors of maternal and cord blood vitamin D levels. A *p*-value of < 0.05 was considered statistically significant.

**Results:**

The median maternal and cord blood serum vitamin D levels were 9.86 [8.32–11.17] ng/mL and 10.12 [8.58–11.01] ng/mL, respectively. Vitamin D concentrations were below 20 ng/mL in 99.7% of maternal blood samples and 99.5% of cord blood samples. Only 76 women (19.1%) reported vitamin D supplementation during pregnancy, with a mean daily intake of 1,052.9 international units. Maternal and cord blood serum vitamin D levels were positively associated (Spearman’s rho = 0.357, CI: 0.267–0.440, *p* < 0.001). Maternal serum vitamin D levels were higher among women who delivered in summer than among those who delivered in winter (11.76 [10.87–12.41] ng/mL vs. 9.39 [7.71–10.19] ng/mL, CI: 1.845–3.133, *p* < 0.001). Cord blood serum vitamin D levels were also higher among neonates born in summer than among those born in winter (10.65 [10.14–11.42] ng/mL vs. 9.63 [8.02–10.74] ng/mL, CI: 0.441–2.023, *p* = 0.002) and lower in small-for-gestational-age (SGA) neonates than in non-SGA neonates (9.3 [6.70–10.51] ng/mL vs. 10.19 [8.76–11.05] ng/mL, CI: −2.124 to −0.018, *p* = 0.046).

**Conclusion:**

Vitamin D insufficiency was highly prevalent among pregnant women and newborns in this Jordanian cohort. Further evaluation of targeted screening, appropriate supplementation strategies, and public health interventions is warranted.

**Clinical trial registration:**

ClinicalTrials.gov, Identifier NCT04944212.

## Introduction

Poor maternal vitamin D status has been associated with adverse pregnancy outcomes, including gestational diabetes mellitus, preeclampsia, and the birth of infants who are small for gestational age (1–3). In addition, vitamin D deficiency may have long-term effects on fetal bone development, immune function, and neuropsychological development (4). Vitamin D status in adults is typically assessed by measuring serum 25-hydroxyvitamin D (25[OH]D) concentrations. However, the optimal serum 25[OH]D concentration remains a topic of debate. The National Academy of Medicine defines vitamin D insufficiency as a 25[OH]D concentration between 12 and <20 ng/mL (30–50 nmol/L) and vitamin D deficiency as a level of <12 ng/mL (<30 nmol/L) (4). However, the ideal serum 25[OH]D level during pregnancy remains unclear (5).

Globally, vitamin D insufficiency is a widespread concern among pregnant women, with prevalence rates varying based on region, diet, lifestyle, and sunlight exposure. For example, a cross-sectional study from Germany reported that 77% of pregnant women had low vitamin D levels, defined as serum 25(OH)D concentrations below 20 ng/mL (6). Similar findings have been reported in the Middle East. A study conducted in Lebanon found that 63% of pregnant women had vitamin D levels below 20 ng/mL (7). Similarly, a study of 221 pregnant women in Saudi Arabia showed that 46.5% had vitamin D insufficiency, while 13.6% had vitamin D deficiency (8).

In Jordan, a study of non-pregnant women of reproductive age found that 60.3% had vitamin D levels below 12 ng/mL. The prevalence of low vitamin D was significantly higher among women living in urban areas and among those who wore a scarf, hijab, or niqab (9). Another study by El-Khateeb et al. (*n* = 4,056) reported that the prevalence of vitamin D insufficiency, defined as serum 25(OH)D levels of <20 ng/mL, was 54% in male individuals and 78.5% in female individuals in Jordan (10). Khuri-Bulos et al. (*n* = 3,512) found a high prevalence of vitamin D insufficiency among newborns in Amman, with 94.1% having cord blood 25(OH)D levels of <20 ng/mL, even among those born during the spring and summer months (11). Lower gestational age, maternal smoking, winter births, and lack of vitamin D supplementation were associated with lower cord blood vitamin D levels (11). To date, no studies have evaluated vitamin D status among pregnant women in Jordan. Therefore, this study aimed to measure serum 25[OH]D concentrations in Jordanian pregnant women and their newborns and investigate factors associated with maternal and cord blood 25[OH]D levels.

## Materials and methods

### Study population

This cross-sectional study was conducted at the Department of Obstetrics and Gynecology, King Abdullah University Hospital (KAUH), a tertiary referral teaching hospital in Jordan. The study included pregnant women with singleton live fetuses at 34 weeks of gestation or above who attended the labor ward between November 2021 and July 2022. Pregnant women with chronic medical conditions were excluded to reduce potential confounding, as such conditions may independently influence maternal vitamin D status and pregnancy outcomes. Written informed consent was obtained from all participants. The study was approved by the KAUH Institutional Review Board (Approval No. 131/2020) and was conducted in accordance with the ethical principles outlined in the Declaration of Helsinki. This study was registered with ClinicalTrials.gov (NCT04944212).

Data were collected by registrars in the labor ward and included maternal age, parity, weight, height, and antenatal complications during the current pregnancy. Additional data included gestational age; mode and date of delivery; neonatal weight, length, and sex; Apgar scores at 1 and 5 min; umbilical cord arterial pH; and daily sunlight exposure, defined as time spent outdoors. Information on antenatal vitamin D supplementation was also collected, including supplement use, reported daily dose, and duration of supplementation. To better estimate total supplementation exposure, cumulative vitamin D dose was calculated by multiplying the reported daily dose by the duration of supplementation in days. The cumulative vitamin D dose was expressed as the total international units (IU) received during pregnancy. Body mass index (BMI) was calculated as weight in kilograms divided by height in meters squared. Season of delivery was classified according to the month of birth. Deliveries occurring from November to March were categorized as winter deliveries, while those occurring from June to July were categorized as summer deliveries, based on the study recruitment period in Jordan.

Antenatal complications were defined as follows: small for gestational age (SGA) was defined as a birth weight below the 10th percentile for gestational age, while large for gestational age (LGA) was defined as a birth weight greater than the 90th percentile for gestational age (12). Gestational diabetes mellitus (GDM) was diagnosed between 24 and 28 weeks of gestation using a 100-g oral glucose tolerance test (OGTT) when two or more of the following threshold values were met or exceeded: fasting glucose level of ≥5.3 mmol/L, 1-h glucose level of ≥10.0 mmol/L, 2-h glucose level of ≥8.6 mmol/L, or 3-h glucose level of ≥7.8 mmol/L (13). Gestational hypertension (GHTN) was defined by the new onset of hypertension, systolic blood pressure ≥ 140 mmHg, and/or diastolic blood pressure ≥ 90 mmHg at or after 20 weeks of gestation, in the absence of proteinuria or new signs of end-organ dysfunction (14).

### Sample collection and analysis

A total of 396 pregnant women participated in the study. Umbilical cord blood samples were collected by qualified staff nurses from the umbilical cord vein immediately after clamping. For mothers who had vaginal deliveries, maternal blood samples were collected just before delivery. For mothers who underwent elective or emergency cesarean sections, maternal samples were collected immediately before surgery. All samples were collected in serum separator tubes (SSTs) and centrifuged for 10 min at 2,000×*g*. The resulting serum was separated, divided into aliquots, and stored at −80 °C until analysis. Serum 25[OH]D levels were measured using an enzyme-linked immunosorbent assay (ELISA), according to the manufacturer’s protocols (MyBioSource, San Diego, CA, USA).

### Statistical analysis

Statistical analyses were performed using SPSS software, version 26 (IBM Corp., Armonk, NY, USA). The distribution of continuous variables was assessed using the Kolmogorov–Smirnov test. Qualitative variables are presented as frequencies and percentages, while quantitative variables are presented as medians and interquartile ranges (IQRs), as appropriate. Differences between groups for continuous variables were assessed using the Mann–Whitney U test. Spearman’s correlation test was used to evaluate associations between continuous variables.

Variables with *p*-values of <0.25 in the univariate analysis and/or variables considered clinically relevant were considered for inclusion in the multivariable linear regression analysis. A stepwise multivariable linear regression was used to identify the final independent predictors of maternal and cord blood serum vitamin D levels. To assess the consistency of the findings, an enter-method multivariable linear regression model including the selected variables was also performed. Multicollinearity was assessed using tolerance values, and model performance was evaluated using adjusted *R*^2^. Regression results are presented as unstandardized coefficients, standardized beta coefficients, standard errors, *p*-values, 95% confidence intervals, and adjusted *R*^2^ values. A *p*-value of less than 0.05 was considered statistically significant.

## Results

### Study population

A total of 396 mothers and their newborns were included in the study. Their baseline characteristics are summarized in Table 1. The mean maternal age was 31.3 ± 0.3 years, and the mean BMI was 30.2 ± 0.2 kg/m^2^. Antenatal complications were reported in 111 women (28%), with small for gestational age (SGA) being the most common complication (10.4%). The mean gestational age at delivery was 38 ± 0.07 weeks, and the mean birth weight was 3.1 ± 0.02 kg. The most common mode of delivery was vaginal delivery (74%). The majority of newborns (91.7%) had Apgar scores ≥ 8, and 34 newborns (8.6%) were admitted to the neonatal intensive care unit. Only 76 women (19.1%) reported vitamin D supplementation during pregnancy, with a mean daily intake of 1052.9 international units (IU). The mean daily sunlight exposure among pregnant women was 53.2 ± 3.5 min.

**Table 1:** Baseline maternal and neonatal demographic and clinical characteristics of the study cohort (*N* = 396).

Variables	Mean ± SE
	Frequency (%)
Maternal characteristics	
Age (calendar year)	31.3 ± 0.3
BMI on admission (kg/m^2^)	30.2 ± 0.2
Parity	
Nulliparous	65 (16.4)
Multiparous	331 (83.6)
Miscarriages	
<3 miscarriages	371 (93.7)
≥3 miscarriages	25 (6.3)
Reported antenatal complications	
Small for gestational age	41 (10.4)
Large for gestational age	24 (6.1)
Gestational diabetes mellitus	20 (5.1)
Preterm premature rupture of membrane	10 (2.5)
Premature rupture of membrane	9 (2.3)
Gestational hypertension	7 (1.8)
Cholestasis of pregnancy	1 (0.3)
Antenatal use of vitamin D3 supplements	76 (19.1)
Antenatal vitamin D3 intake from supplements (IU/day) (*N* = 76)	1,052.9 ± 63.9
Daily sunlight exposure (minutes)	53.2 ± 3.5
Mode of delivery	
Vaginal delivery	293 (74)
Cesarean section	103 (26)
Season of delivery	
Summer	153 (38.6)
Winter	243 (61.4)
Neonatal characteristics	
Sex	
Male	199 (50.3)
Female	197 (49.7)
Gestational age (weeks)	38 ± 0.07
Birth weight (kg)	3.1 ± 0.02
Birth length (cm)	49.8 ± 0.2
Head circumference (cm)	35 ± 0.1
Apgar score at 1 min	8 ± 0.03
Apgar score at 5 min	9 ± 0.02
Umbilical cord arterial pH (*N* = 241)	7.3 ± 0.004
Neonatal intensive care unit admission	34 (8.6%)

SE, standard error of the mean; BMI, body mass index; IU, international unit.

### Maternal and cord blood vitamin D concentrations

The median maternal and cord blood serum vitamin D levels were 9.86 [8.32–11.17] ng/mL and 10.12 [8.58–11.01] ng/mL, respectively (Table 2). Using a serum 25[OH]D level of <20 ng/mL as the cutoff for vitamin D insufficiency, 99.7% of maternal blood samples and 99.5% of cord blood samples had insufficient vitamin D concentrations. In addition, 52.8% of maternal vitamin D concentrations and 47% of cord blood vitamin D concentrations were below 10 ng/mL.

**Table 2:** Maternal and cord blood serum vitamin D concentrations.

Vitamin D level	Median [IQR]	Mean ± SE
Maternal vitamin D (ng/mL)	9.86 [8.32–11.17]	9.62 ± 0.15
Cord blood vitamin D (ng/mL)	10.12 [8.58–11.01]	9.62 ± 0.16

IQR, interquartile range; SE, standard error of the mean. A significant positive correlation was found between maternal and cord blood serum vitamin D levels using Spearman’s correlation test (Spearman’s rho = 0.357, 95% CI: 0.267–0.440, *p* < 0.001).

Maternal serum vitamin D levels were significantly higher among women who delivered in summer than among those who delivered in winter (11.76 [10.87–12.41] ng/mL vs. 9.39 [7.71–10.19] ng/mL, CI: 1.845–3.133, *p* < 0.001). A significant positive association was found between maternal serum and cord blood vitamin D levels (Spearman’s rho = 0.357, CI: 0.267–0.440, *p* < 0.001). Furthermore, cord blood serum vitamin D levels were significantly higher among infants born in summer than among those born in the winter (10.65 [10.14–11.42] ng/mL vs. 9.63 [8.02–10.74] ng/mL, CI: 0.441–2.023, *p* = 0.002) and significantly lower among SGA infants than among non-SGA infants (9.3 [6.70–10.51] ng/mL vs. 10.19 [8.76–11.05] ng/mL, CI: −2.124–0.018, *p* = 0.046).

The univariate analysis showed a significant association between maternal vitamin D concentrations and antenatal vitamin D3 supplementation; however, this association was not retained in the multivariable linear regression model. No correlation was found between vitamin D status and time spent outdoors. Other variables did not show significant associations with maternal and/or cord blood vitamin D levels (Tables 3, 4).

**Table 3:** Univariate analysis of variables significantly associated with maternal and/or cord blood serum vitamin D levels (*N* = 396).

Variable	Median [IQR]	*p*-value
Maternal vitamin D		
Season of delivery		
Summer	11.76 [10.87–12.41]	<0.001
Winter	9.39 [7.71–10.19]	
Antenatal use of vitamin D3 supplements		
Yes	10.62 [8.81–12.09]	0.004
No	9.84 [8.22–10.87]	
Miscarriages		
<3 miscarriages	9.82 [8.22–11.10]	0.003
≥3 miscarriages	10.56 [10.21–11.60]	
Gestational hypertension		
Yes	12.06 [10.65–12.62]	0.003
No	9.86 [8.72–11.12]	
Cord blood vitamin D		
Season of delivery		
Summer	10.65 [10.14–11.42]	<0.001
Winter	9.63 [8.02–10.74]	
Small for gestational age		
Yes	9.30 [6.70–10.51]	0.017
No	10.19 [8.76–11.05]	
Neonatal intensive care unit admission		
Yes	9.86 [8.55–10.42]	0.030
No	10.21 [8.59–11.10]	

IQR, interquartile range. Two-sided *p*-values were calculated using the Mann–Whitney U test. Only variables that showed statistically significant associations in the univariate analysis are presented.

**Table 4:** Final predictors independently associated with maternal and cord blood serum vitamin D levels in stepwise multivariable linear regression analysis (*N* = 396).

Predictor	Unstandardized coefficients (*B*)	Standardized coefficients (*β*)	Standard error	*p*-value	95% CI
Maternal vitamin D					
Delivery in summer	2.489	0.372	0.328	<0.001	1.845–3.133
Cord blood vitamin D					
Delivery in summer	1.232	0.177	0.402	0.002	0.441–2.023
Maternal vitamin D	0.202	0.157	0.074	0.007	0.056–0.348
SGA	−1.071	−0.101	0.535	0.046	−2.124 to −0.018

*B*, unstandardized regression coefficient; *β*, standardized regression coefficient; CI, confidence interval; SGA, small for gestational age. This table presents the final predictors retained in the stepwise multivariable linear regression model. Adjusted *R*^2^ was 0.138 for the maternal vitamin D model and 0.080 for the cord blood vitamin D model.

## Discussion

There is growing evidence linking low maternal vitamin D status to adverse pregnancy outcomes, including gestational hypertension, preeclampsia, gestational diabetes, preterm birth, and low birth weight (1–3, 15, 16). Furthermore, gestational vitamin D insufficiency has been associated with several neonatal and long-term health outcomes, including impaired bone development, immune-related disorders, asthma, and neurodevelopmental outcomes (4, 17, 18). Pregnant women in regions such as Asia, the Middle East, Africa, and Latin America are particularly at risk of vitamin D insufficiency, with prevalence rates in these areas being among the highest globally (15). In the Middle East, the high rates of vitamin D insufficiency among pregnant women may be attributed to several regional, dietary, lifestyle, and cultural factors, including limited outdoor activity, clothing practices, low dietary vitamin D intake, inadequate supplementation, obesity, genetic factors, and medical conditions such as hyperglycemia or malabsorption syndromes (19). Our findings are consistent with this regional pattern, as vitamin D insufficiency was highly prevalent among both pregnant women and their newborns in this Jordanian cohort.

During pregnancy, the fetus receives vitamin D through placental transfer, and fetal vitamin D status is largely dependent on maternal vitamin D status (15, 20). A strong positive correlation between maternal and cord blood 25[OH]D concentrations has been demonstrated in several studies, including the present study (15). In our cohort, lower cord blood vitamin D levels were observed among SGA infants compared with non-SGA infants. This finding is consistent with studies suggesting an association between low maternal vitamin D status and impaired fetal growth or low birth weight (1, 3, 15, 16). However, due to the cross-sectional design of our study, this finding should be interpreted as an association rather than evidence of causality. Fetal growth is influenced by multiple maternal, placental, nutritional, genetic, and environmental factors, many of which were not fully assessed in the present study.

In our study, vitamin D insufficiency was more prevalent among neonates born during the winter months compared to those born in summer. This seasonal variation has been reported in several studies (6, 16, 18). However, the sunny season did not appear to protect newborns from vitamin D insufficiency, suggesting that sunlight exposure alone may be insufficient to maintain adequate vitamin D status in this population.

In our cohort, only 19.1% of women reported taking vitamin D supplements during pregnancy. Although supplement users had higher maternal serum vitamin D levels in the univariate analysis, this association was not retained in the multivariable regression model. This finding should be interpreted cautiously, as supplementation was reported by only a small proportion of women, and the reported supplementation dose was relatively low. In addition, although information on dose and duration was collected, other factors such as adherence, timing of initiation during pregnancy, baseline vitamin D status, and dietary vitamin D intake may have influenced the observed effect of supplementation. Previous studies, including randomized trials, have shown that vitamin D supplementation can increase maternal 25(OH)D levels in a dose-dependent manner, with higher doses producing greater increases in vitamin D concentrations (21).

There is ongoing debate regarding routine vitamin D supplementation and screening during pregnancy. The National Academy of Medicine recommends a daily allowance of 600 IU of vitamin D for all reproductive-age women, including during pregnancy and lactation (22). The American College of Obstetricians and Gynecologists (ACOG) recommends routine supplementation with the dose provided in standard prenatal vitamins until further evidence supports an alternative dosage (23). For pregnant women with vitamin D deficiency, the ACOG states that 1,000–2,000 IU/day of vitamin D is generally considered safe, although the safety of higher-dose regimens has not been adequately studied (23). Similarly, there is no consensus on universal vitamin D screening during pregnancy. A survey in the Asia-Pacific region found that the majority of healthcare facilities lack national guidelines for vitamin D screening during pregnancy (24). The ACOG recommends screening only high-risk women, citing insufficient evidence to support universal screening (23). Targeted supplementation based on risk factors, such as darker skin, obesity, limited sun exposure, and other lifestyle or cultural factors, has been proposed as a potentially more cost-effective approach (25).

Given the high prevalence of vitamin D insufficiency observed in our study population, further studies are needed to evaluate the potential role of targeted screening and appropriately designed supplementation strategies among pregnant women in Jordan. In addition, public health strategies aimed at improving vitamin D status, such as health education programs and food fortification, may be considered. The Food-Based Solutions for Optimal Vitamin D Nutrition and Health Through the Life Cycle (ODIN) project demonstrated that fortifying commonly consumed foods, such as dairy products, with vitamin D may be an effective strategy to increase population-level vitamin D status, particularly among high-risk groups (26).

### Study limitations

Our study has several limitations that should be considered. First, vitamin D status was assessed only at the end of pregnancy; therefore, 25[OH]D concentrations during the first and second trimesters were not evaluated. Second, recall bias may have affected the reported use of vitamin D supplements and outdoor activity during pregnancy. Third, due to its cross-sectional design, this study can identify associations between maternal and cord blood vitamin D levels and pregnancy or neonatal characteristics, but it cannot establish causality. Fourth, although information on vitamin D supplementation use, reported daily dose, and duration of supplementation was collected, data on the exact timing of initiation during pregnancy and adherence/compliance were not available. This may have limited our ability to fully evaluate the effect of supplementation on maternal and cord blood vitamin D levels.

Another limitation is that several potential confounding factors that may influence vitamin D status were not fully evaluated. Although data on vitamin D supplementation and daily sunlight exposure were collected, detailed information on dietary vitamin D intake, socioeconomic status, physical activity, and maternal education level was not available. Clothing style was not included in the analysis; however, the majority of women in the study cohort wore hijab, limiting variability and the ability to assess its independent effect on vitamin D status. In addition, the unequal number of participants recruited across seasons may have affected the precision of seasonal comparisons. These unmeasured factors may have influenced maternal and cord blood vitamin D levels and should be considered in future studies.

## Conclusion

Vitamin D insufficiency was highly prevalent among pregnant women and their newborns in this Jordanian cohort. Cord blood vitamin D levels were positively associated with maternal vitamin D levels and were lower among SGA infants, suggesting a possible association between vitamin D status and fetal growth. Although vitamin D supplementation was associated with higher maternal vitamin D levels in the univariate analysis, this association was not retained in the multivariable model. This finding should be interpreted cautiously because only a small proportion of women reported vitamin D supplementation, and the average reported dose was relatively low. Targeted screening of high-risk women, appropriately designed supplementation strategies, and public health interventions such as education and food fortification may help improve vitamin D status in this population.

## Data Availability

The datasets presented in this article are not readily available because the datasets used and/or analyzed during the current study are available from the corresponding author upon reasonable request. Requests to access the datasets should be directed to obeidat_rawan@yahoo.com.
